# Triclosan Disrupts SKN-1/Nrf2-Mediated Oxidative Stress Response in *C*. *elegans* and Human Mesenchymal Stem Cells

**DOI:** 10.1038/s41598-017-12719-3

**Published:** 2017-10-03

**Authors:** Dong Suk Yoon, Yoorim Choi, Dong Seok Cha, Peng Zhang, Seong Mi Choi, Mohammad Abdulmohsen Alfhili, Joseph Ryan Polli, DeQwon Pendergrass, Faten A. Taki, Brahmam Kapalavavi, Xiaoping Pan, Baohong Zhang, T. Keith Blackwell, Jin Woo Lee, Myon-Hee Lee

**Affiliations:** 1 Department of Internal Medicine, Brody School of Medicine at East Carolina University, Greenville, NC 27834 USA; 2 Department of Orthopaedic Surgery, Yonsei University College of Medicine, Seoul, 120-752 South Korea; 3 Brain Korea 21 PLUS Project for Medical Sciences, Yonsei University College of Medicine, Seoul, 120-752 South Korea; 4 Joslin Diabetes Center, One Joslin Place, Boston, MA 02215 USA; 5 Department of Genetics and Harvard Stem Cell Institute, Harvard Medical School, Boston, MA 02115 USA; 6 Department of Clinical Laboratory Sciences, College of Applied Medical Sciences, King Saud University, Riyadh, 11433 Saudi Arabia; 7 Department of Biology, East Carolina University, Greenville, NC 27858 USA; 8 Department of Chemistry, East Carolina University, Greenville, NC 27858 USA; 9 Lineberger Comprehensive Cancer Center, University of North Carolina-Chapel Hill, Chapel Hill, NC 27599 USA; 10 Present Address: Department of Oriental Pharmacy, College of Pharmacy, Woosuk University, Jeonbuk, 565-701 Republic of Korea

## Abstract

Triclosan (TCS), an antimicrobial chemical with potential endocrine-disrupting properties, may pose a risk to early embryonic development and cellular homeostasis during adulthood. Here, we show that TCS induces toxicity in both the nematode *C. elegans* and human mesenchymal stem cells (hMSCs) by disrupting the SKN-1/Nrf2-mediated oxidative stress response. Specifically, TCS exposure affected *C. elegans* survival and hMSC proliferation in a dose-dependent manner. Cellular analysis showed that TCS inhibited the nuclear localization of SKN-1/Nrf2 and the expression of its target genes, which were associated with oxidative stress response. Notably, TCS-induced toxicity was significantly reduced by either antioxidant treatment or constitutive SKN-1/Nrf2 activation. As Nrf2 is strongly associated with aging and chemoresistance, these findings will provide a novel approach to the identification of therapeutic targets and disease treatment.

## Introduction

Triclosan (2,4,4′-trichloro-2′-hydroxydiphenyl ether; TCS) is a broad-spectrum antimicrobial agent that is commonly used in personal care products (e.g., antiseptic soaps, toothpastes, fabrics, and plastics) and medical devices (e.g., surgical sutures, catheters, and ureteral stents)^1–3^. Evidence from *in vitro* and *in vivo* animal studies has linked TCS to numerous human health problems, including allergies, asthma, and eczema in children, as well as breast cancer and neurodegenerative diseases in adults, through various modes of action^4^.

TCS exerts its antimicrobial effect by interfering with enoyl-acyl carrier protein reductase (FabI) activity, which is required for fatty acid and biotin biosynthesis. This ultimately leads to the suppression of bacterial growth. Also, TCS destabilizes bacterial membrane by inducing K^+^ leakage^5^. Importantly, TCS bears a structural similarity to the thyroid hormone thyroxine (T4) and to other known endocrine disruptors, including polychlorinated biphenyls, diethylstilbestrol, and bisphenol A^6^. The aromatic nature and high chlorine content of TCS confer resistance to degradation and persistence in the environment^4^. Similarly, a pharmacokinetic study determined that significant levels of TCS were widely detected in human body fluids and in the surrounding environment^4^. Recent studies have also shown that TCS may interfere with thyroid hormone metabolism in humans by acting as a potential endocrine-disrupting chemical (EDC)^7^. TCS was shown to induce thyroid hormone clearance, possibly through the activation of nuclear receptors such as pregnane X receptor (PXR) and constitutive androstane receptor (CAR). Activated receptors eventually lead to hypothyroxinaemia (significant reduction in serum thyroxine levels) – a hallmark of overt hypothyroidism^4,8,9^. Furthermore, TCS was reported to inhibit the detoxification of foreign chemicals in the body through the interaction with the same nuclear receptors, inducing the expression of hepatic cytochrome P450 2B10 (CYP2b10)^10^. *In vivo* studies in mouse models have shown that TCS disrupted liver integrity and compromised liver function^10^. Moreover, mice subjected to TCS were more susceptible to chemically induced liver tumors^10^. Based on these investigations and the potential risk associated with TCS exposure, it is currently classified as a category III compound by the FDA^1^, which has recently banned its use in antibacterial soaps. However, the underlying mechanisms by which TCS causes these numerous health problems at the molecular and cellular levels remain poorly understood.

Nuclear factor erythroid-2-related factor 2 (Nrf2) is a mammalian, basic leucine zipper transcription factor involved in the activation of more than 100 genes responsible for cellular stress response and cytoprotection^11^. The antioxidant response elements (AREs) are regulatory DNA sequences common to Nrf2 downstream target genes. Upon induction, Nrf2 dissociates from its cytoplasmic scaffold protein, Keap1, and translocates to the nucleus where it activates a plethora of genes governing energy metabolism, reactive oxygen species (ROS) scavenging and elimination, anti-inflammatory signalling, and xenobiotic detoxification^12–14^. SKN-1 (SKiNhead-1) is the *C. elegans* homolog of Nrf2, and both proteins share the conserved function of cellular defense in response to oxidative stress and xenobiotics^15,16^. We here show that TCS induces toxicity, at least in part, by disrupting the SKN-1/Nrf2-mediated oxidative stress response. In this study, we used two powerful model systems, the nematode *C. elegans* and human mesenchymal stem cells (hMSCs). *C. elegans* has emerged as an attractive model system for the functional analysis of various bioactive and natural compounds. hMSCs are currently used in many clinical trials and regenerative medicine studies for their multipotency and self-renewal capabilities. Since the mechanism of action of TCS in SKN-1/Nrf2-mediated oxidative stress response may be conserved in diverse cellular processes during animal development, tissue homeostasis, and disease modelling, our findings represent seminal insights on the interaction of TCS with host cells and the role of SKN-1/Nrf2 in xenobiotic stress response.

## Results

### Long-term TCS treatment perturbs survival of wild-type worms

**Figure 1:**
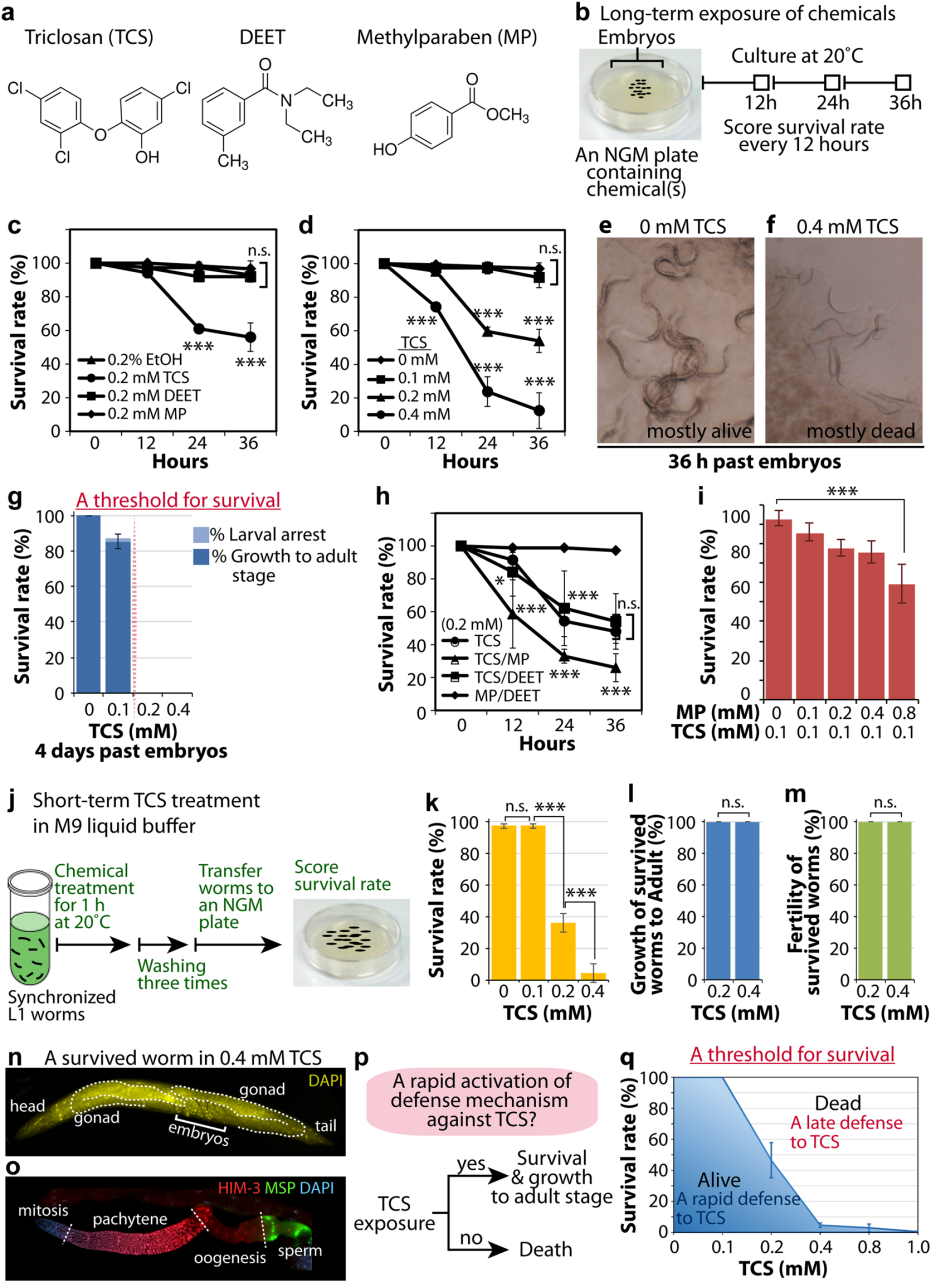
Effects of potential endocrine disrupting chemicals on wild-type worm survival. (**a**) Chemical structures of potential endocrine disrupting chemicals (EDCs): triclosan (TCS), DEET, and methylparaben (MP). (**b**) Strategy for long-term treatment of ECDs on NGM plates. (**c** and **d**) Individual effects of EDCs on the survival of wild-type worms during the first 36 hours from the embryo stage. (**e** and **f**) DIC pictures of wild-type worms treated with EtOH control (0 mM TCS) and 0.4 mM TCS. (**g**) The survival rate of TCS-treated wild-type worms and the percentage of worms that reached adult stage at 4 days past embryos. A red line indicates the potential threshold for the survival of wild-type worms against TCS. (**h**) The combinational effect of EDCs (0.2 mM each) on the survival of wild-type worms over the first 36 hours from the embryo stage. (**i**) Dose-dependent effect of MP on the survival rate of 0.1 mM TCS-treated wild-type worms. (**j**) Strategy for short-term TCS treatment in M9 liquid buffer. (**k**) The survival rate of synchronized L1 wild-type worms. (**l**) The percentage of wild-type worms that reached adult stage against TCS. (**m**) The percentage of worms that have fertile gonads against TCS. (**n**) DAPI staining of a worm that survived in 0.4 mM TCS. (**o**) Survived adult hermaphrodite germlines were extruded and co-stained with anti-HIM-3 antibody (meiosis marker) anti-MSP antibody (sperm marker), and DAPI (DNA). (**p**) A rapid activation of defense mechanism against TCS contributes to the survival of wild-type worms and their growth to adult stage without obvious developmental defects. (**q**) The potential threshold for the survival of wild-type worms against TCS. Standard deviation bars were calculated from at least three independent experiments. *p* < 0.05(*); *p* < 0.01(**); *p* < 0.001(***); Not statistically significant (n.s.).

At certain doses, EDCs can interfere with the endocrine system and result in developmental disorders. We specifically focused on three potential EDCs, namely triclosan (TCS), *N*,*N*-diethyl-*meta*-toluamide (DEET), and methylparaben (MP) (Fig. 1a). TCS is an antimicrobial compound found in consumer products and medical devices^1–3^, DEET is a highly effective insect repellent and has been widely used for several decades^17^, and methylparaben is commonly used as a preservative in cosmetics and foods^18,19^. The toxic effects of these potential EDCs were examined using the nematode *C. elegans* as a model system. To observe the effect of EDCs on the survival of wild-type worms (from early L1 larvae to young adults), isolated embryos were placed on NGM plates containing 0.2 mM TCS, 0.2 mM DEET, 0.2 mM MP, or 0.2% ethanol (EtOH), which was used as a control, and incubated for 36 hours at 20 °C (Fig. 1b). The survival rate was then determined every 12 hours by scoring live and dead worms under a dissecting microscope. While the effects of DEET and MP on the survival of wild-type worms were comparable to those of the EtOH control, TCS significantly decreased the survival rate of wild-type worms dose-responsively (Fig. 1c,d). Resistant, live worms showed pharyngeal pumping, whereas sensitive, dead worms appeared rod-like in shape and slightly uneven in texture (Fig. 1e,f). We also scored the survival rates 4 days past the embryo stage. TCS treatment at 0.1 mM slightly decreased the survival rate of wild-type worms, whereas no worms survived 0.2 and 0.4 mM exposure levels (Fig. 1g). We next examined the ability of long-term TCS treatment to exert developmental defects in wild-type worms. Notably, the majority of surviving worms at 0.1 mM TCS reached adult stage and showed normal fertility (Fig. 1g). This result suggests that 0.1 mM TCS seems to be a potential threshold concentration for survival. Next, we investigated the synergistic effect of TCS and either 0.2 mM DEET or 0.2 mM MP combined treatment on the survival of wild-type worms. The co-exposure of DEET and MP did not affect the survival rate of wild-type worms (Fig. 1h). However, coupled with TCS treatment, MP significantly exacerbated survival in a dose-dependent manner (Fig. 1h,i). Taken together, our results suggest that long-term TCS exposure perturbs survival of wild-type worms, and that TCS toxicity was aggravated by co-treatment with MP.

### Short-term TCS treatment also perturbs survival of wild-type worms

We also tested the short-term effect of TCS exposure on the survival rate of wild-type worms. L1 synchronized wild-type worms were subjected to TCS and EtOH control in M9 liquid buffer for 1 hour at 20 °C and the survival rates were scored (Fig. 1j). Notably, TCS treatment significantly decreased the survival of the wild-type worms in a dose-dependent fashion (Fig. 1k; see Supplementary Fig. 1). Estimates of TCS daily intake reveal concentrations of 47, 65, and 73 ug/kg/day, for men, women, and children, respectively^2^. Also, levels as high as 2,100 ug/kg lipid weight and 3790 ug/L were detected in human breast milk^20^, and urine^21^, respectively. Although the effective concentration of TCS was higher than environmentally relevant levels, in toxicological paradigm, it often requires higher concentrations of drugs to achieve bioavailability. Also, *C. elegans* has an outer cuticle that protects against the influx of chemicals into the organism^22,23^. This renders the nematode more resistant to chemical exposure, necessitating high testing concentrations. Consistent with this notion, mutations in cuticle proteins (e.g., collagen) cause hypersensitivity to EDCs in *C. elegans* ^22^. Exposure levels in the study were chosen to facilitate the identification of molecular mechanisms associated with TCS treatment in a relatively short period of time, as opposed to the low concentrations found in the environment which would typically require long, repeated exposure incidents for a biological response to ensue.

Next, to assess the inter-stage variation in response to TCS, the survival rate of adult worms was also measured. The results showed that TCS decreased the survival of adult worms by a similar magnitude (see Supplementary Fig. 2). Remarkably, worms that survived the short-term treatment reached adult stage and were fecund (Fig. 1l–n). We also examined germline development by staining dissected gonads with anti-HIM-3 (meiotic cell marker), anti-MSP (Major Sperm Protein, sperm marker), and DAPI (DNA marker). Germline staining results indicated that survived worms likely had normal germline proliferation, differentiation, and gametogenesis (Fig. 1o). Collectively, these results suggest that (1) TCS dose-dependently perturbs the survival of wild-type worms; and (2) even at higher concentration of TCS, some worms survived and reached adult stage without obvious developmental defects. Therefore, in light of these findings, we proposed that defense mechanisms in survived worms were activated immediately upon TCS exposure (Fig. 1p) to levels sufficient to confer resistance and survival (Fig. 1q).

### TCS increases the level of intracellular ROS

**Figure 2:**
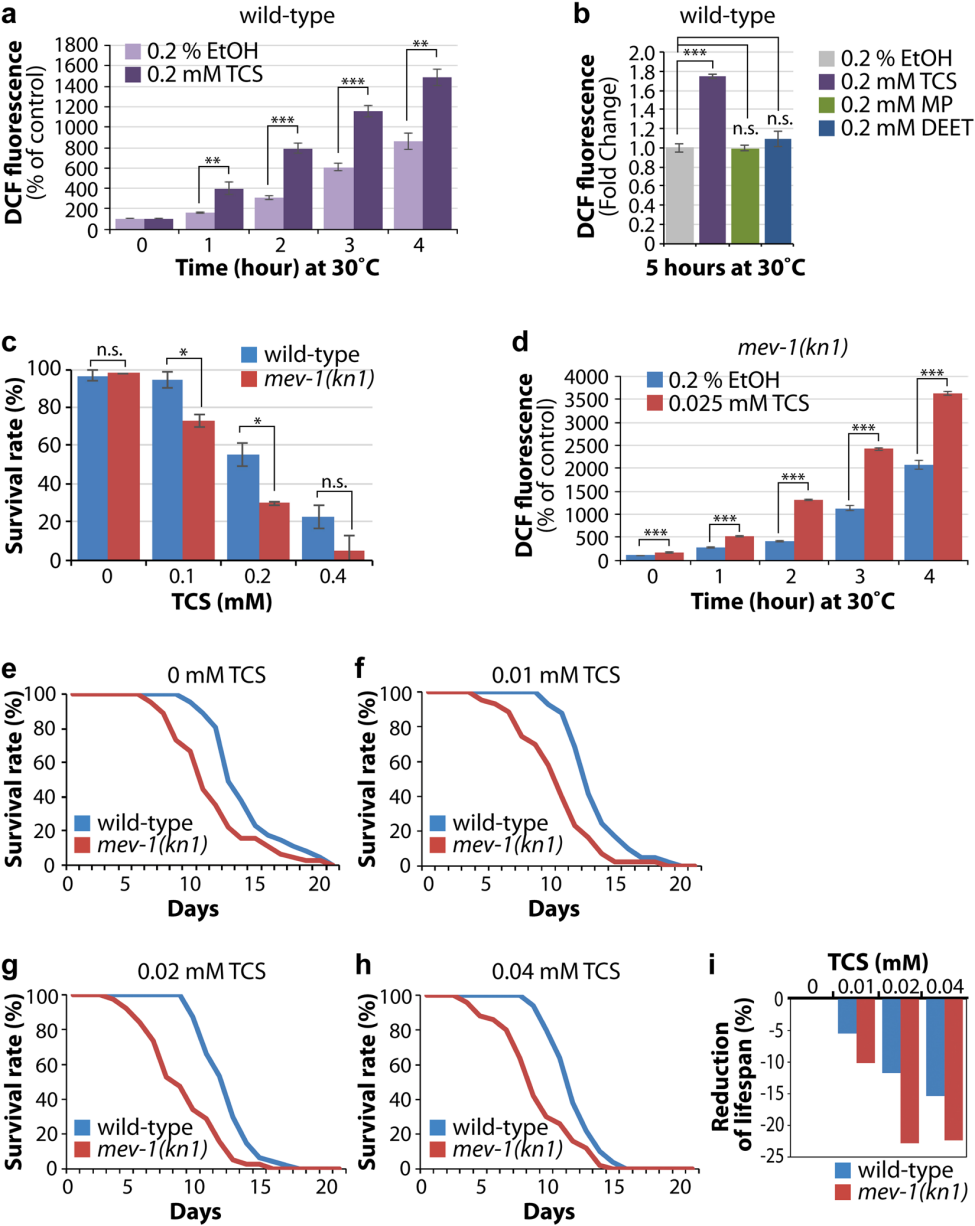
Individual and synergistic effects of TCS on the lifespan of worms. (**a**) 0.2 mM TCS exposure significantly increases intracellular ROS level at 30 °C. Intracellular ROS level was measured spectrophotometrically at excitation and emission wavelengths of 485 nm and 535 nm, respectively. (**b**) Fold change of ROS levels in chemically treated worms. Synchronized L1 worms were exposed to 0.2% EtOH and 0.2 mM of TCS, MP, or DEET. Five hours later, intracellular ROS levels were measured as described in materials and methods. For average fold change of ROS levels, DCF fluorescence values of TCS, MP, or DEET-treated worms were divided by those of EtOH control. (**c**) The effect of TCS on the survival of *mev-1(kn1)* mutants. The *mev-1(kn1)* mutants are more sensitive to TCS than wild-type worms. (**d**) Intracellular ROS levels in *mev-1(RNAi)* worms in 0.2% EtOH and 0.025 mM TCS. (**e**–**h**) The lifespan of wild-type and *mev-1(kn1)* mutant worms at concentrations of 0, 0.01, 0.02, and 0.04 mM TCS. (**i**) Normalized average lifespan of wild-type, and *mev-1(kn1)* mutant worms. The graph shows the lifespan reduction of TCS-treated worms compared with non-treated worms. See Supplementary Table 3 for the statistical analysis of lifespan. Standard deviation bars were calculated from at least three independent experiments. *p* < 0.05(*); *p* < 0.01(**); *p* < 0.001(***); Not statistically significant (n.s.).

The potential correlation of TCS with oxidative stress was recently described in rat thymocytes^24^ and *Tigriopus japonicus* ^25^, but the underlying molecular mechanism remains unknown. To assess the degree of oxidative stress caused by TCS exposure, we conducted a time-course experiment to measure the level of intracellular ROS using the molecular probe H_2_DCF-DA in a 96-well plate. Interestingly, exposure to 0.2 mM TCS resulted in a significant increase in the level of intracellular ROS time-dependently (Fig. 2a). We also examined the effect of MP and DEET on intracellular ROS production. No significant ROS formation was detected following 0.2 mM of MP or DEET treatment (Fig. 2b). To determine whether TCS-induced mortality was positively correlated with oxidative stress, we examined the effect of TCS on survival using a *mev-1* (abnormal MEthyl Viologen sensitive-1) mutant, which is sensitive to oxidative stress^26,27^. L1 stage *mev-1(kn1)* mutants were exposed to 0–0.4 mM TCS for 1 hour at 20 °C and their survival rates were determined as described above. Compared to wild-type worms, the survival rate of *mev-1(kn1)* mutants was significantly decreased even at the TCS concentration of 0.1 mM (Fig. 2c). We also compared the levels of ROS in *mev-1(kn1)* mutants by H_2_DCF-DA staining in the absence or presence of TCS. In congruence with the survival rate findings, intracellular ROS levels were significantly increased in the *mev-1(kn1)* mutants even at the TCS concentration of 0.025 mM (Fig. 2d). These results suggest that TCS may affect the survival of *C. elegans*, at least in part, by the induction of oxidative stress.

### TCS exposure reduced the lifespan of *C. elegans*

The disruption of oxidative stress resistance affects lifespan^28^. To determine the influence of TCS on the lifespan of *C. elegans*, we cultured wild-type L1 worms on NGM plates containing low concentrations of TCS (0, 0.01, 0.02, and 0.04 mM) and recorded their survival rates daily under a dissecting microscope. TCS decreased the lifespan of wild-type worms in a dose-dependent manner (Fig. 2e–h; see Supplementary Table 3). We used the *mev-1(kn1)* mutants to test whether the observed lifespan shortening was related to oxidative stress. Mutations in the *mev-1* gene rendered worms hypersensitive to oxidative stress, and led to a slightly decreased lifespan^29^ (Fig. 2e). In spite of this phenotype, TCS treatment was still able to further augment this effect (Fig. 2f–h). To measure the percentage reduction in lifespan, the average lifespan of *mev-1(kn1)* mutants was divided by that of wild-type worms at each concentration of TCS. Even at the low concentration of 0.01 mM, the normalized average lifespan of *mev-1(kn1)* mutants was significantly decreased (Fig. 2i). These results suggest that the TCS-induced decrease in lifespan of wild-type worms involved the induction of oxidative stress.

### TCS altered the expression of genes associated with oxidative stress response

**Figure 3:**
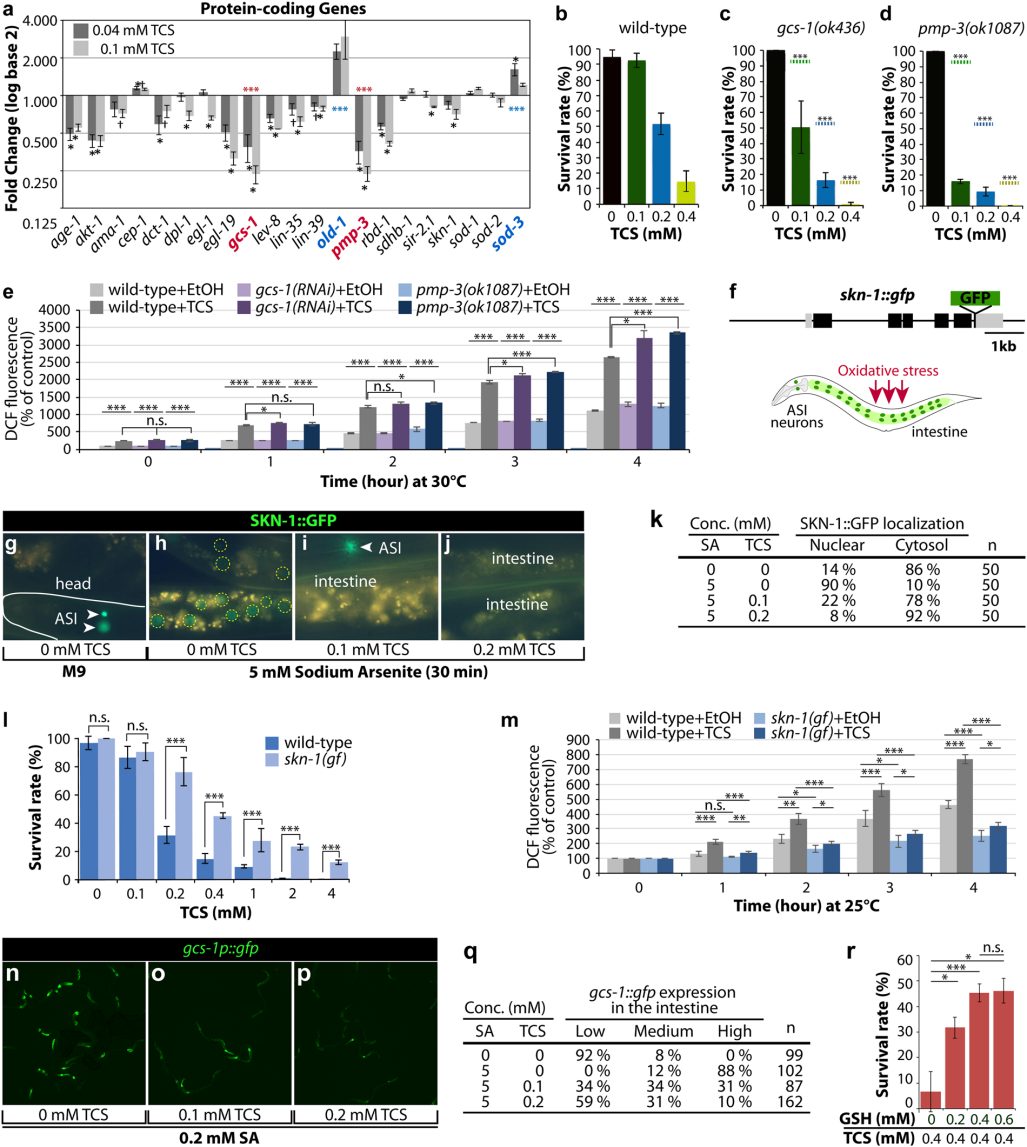
TCS inhibits the nuclear localization of SKN-1 and expression of its target genes. (**a**) Fold change of gene expression for protein-coding genes related to aging, oxidative stress, and detoxification. All fold change values are relative to a control value of one. Values greater than one are considered as upregulation; values less than one are considered as downregulation. Statistical analysis was conducted using ANOVA to determine the significance at *p* < 0.05 (^†^) and *p* < 0.01 (*). See Supplementary Table 4 for the description of the 21 tested genes online. (**b**–**d**) Survival rate of wild-type, *gcs-1(ok436)*, and *pmp-3(ok1087)* mutant worms that were exposed to different concentrations of TCS. Broken dots in c and d indicate the average survival rates of wild-type worms (see Fig. 1b). (**e**) Intracellular ROS level in *gcs-1(RNAi)* and *pmp-3(ok1087)* mutant worms in 0.2% EtOH and 0.05 mM TCS. The levels of DCF fluorescence in *gcs-1(RNAi)* and *pmp-3*(*ok1087*) mutant worms were normalized to those in wild-type worms. (**f**) SKN-1::GFP translational fusion construct that previously rescued maternal *skn-1* lethality^64^. Schematic of SKN-1::GFP expression in ASI neurons and intestinal nuclei under oxidative stress conditions. (**g**–**j**) Larval SKN-1::GFP expression under normal conditions and oxidative stress conditions. (**g**) SKN-1::GFP expression in ASI neurons (arrowheads). (**h**–**j**) SKN-1::GFP localization in the absence or presence of TCS under oxidative stress conditions. (**k**) Fraction of nuclear SKN-1::GFP localization. (**l**) Constitutively activated *skn-1* gain-of-function (gf) mutants are resistant to TCS exposure. (**m**) Intracellular ROS level in *skn-1(gf)* worms in 0.2% EtOH and 0.2 mM TCS. The levels of DCF fluorescence in *skn-1(gf)* worms were normalized to those in wild-type worms. (**n**–**p**) GCS-1::GFP expression in the absence or presence of TCS under oxidative stress conditions. (**q**) The percentages of animals in each expression category are listed. “Low” refers to worms similar to wild-type worms. “Medium” refers to animals in which GCS-1::GFP was apparent at modest levels anteriorly or posteriorly. “High” indicates that GCS-1::GFP was present at high levels both anteriorly and posteriorly as well as detectable throughout the intestine. (**r**) GSH supplementation represses TCS-induced mortality. Standard deviation bars were calculated from at least three independent experiments. *p* < 0.05(*); *p* < 0.01(**); *p* < 0.001(***); Not statistically significant (n.s.).

Chemical compounds can induce physiological effects via stimulation of abnormal gene expression during development^30–32^. To test whether TCS exposure alters the expression of genes associated with oxidative stress response (see Supplementary Table 4), we analysed the fold change in the expression of select stress-response genes using an Eppendorf Mastercycler® Pro PCR machine. The exposure of approximately 2,000 L1 wild-type worms to non-lethal concentrations (0.04 mM and 0.1 mM) of TCS for 2 days at 20 °C led to a significant alteration in gene expression (Fig. 3a). A significant upregulation was only observed in two genes (*old-1* and *sod-3*), while six genes were significantly downregulated (*age-1*, *akt-1*, *egl-19*, *gcs-1*, *pmp-3*, and *rbd-1*); of these, *gcs-1* and *pmp-3* were profoundly downregulated following an increase in TCS concentration (Fig. 3a). These results suggest that TCS may alter the expression of genes that are associated with oxidative stress response and defense mechanisms.

To test whether TCS toxicity relied on *gcs-1* and *pmp-3*, we employed *gcs-1(ok436)* and *pmp-3(ok1087)* single mutant worms. The gene *gcs-1*, which encodes for γ-glutamyl-cysteine synthetase (γGCS), synthesizes glutathione (GSH) and plays a crucial role in the detoxification of oxidants and toxins in cells^33^. The gene *pmp-3* (encoding an ABC transporter) modulates the absorption, metabolism, and cytotoxicity of pharmacological agents^34^. L1 stage wild-type, *gcs-1 (ok436)*, and *pmp-3* (*ok1087*) worms were treated with TCS at concentrations of 0, 0.1, 0.2, and 0.4 mM for 1 hour at 20 °C and the survival rates were measured as described above. The survival rate of wild-type worms was approximately 50% and 15% at 0.2 mM and 0.4 mM TCS, respectively (Fig. 3b), with a significant further decrease in the *gcs-1(ok436)* and *pmp-3(ok1087)* mutants, even at 0.1 mM TCS (Fig. 3c,d). We also compared the levels of intracellular ROS in wild-type, *gcs-1(RNAi)*, and *pmp-3(ok1087)* worms using the molecular probe H_2_DCF-DA in the absence or presence of TCS. Notably, ROS levels in *gcs-1(RNAi)* and *pmp-3(ok1087)* mutant worms were significantly higher than those in wild-type worms even at the lower concentration (0.025 mM) of TCS (Fig. 3e). These results suggest that TCS may induce oxidative stress by altering the expression of genes associated with oxidative stress response.

### TCS inhibits SKN-1 nuclear localization

The major oxidative stress response is controlled by the transcription factor SKN-1, a homologue of the mammalian Nrf2^15^. The transcription of *gcs-1*/γGCS and *pmp-3*/ABC transporter genes is activated by the SKN-1/Nrf2 transcription factor in worms and mice^15,35,36^. The nuclear localization (activation) of SKN-1 proteins increases the expression of detoxification and antioxidant-related genes, which reduce cellular oxidative stress^37,38^. qRT-PCR analysis revealed that the expression of the *skn-*1 gene is slightly subverted in response to TCS (Fig. 3a). It was therefore tested if TCS could inhibit the nuclear localization of SKN-1 protein and the subsequent expression of its target genes. For this purpose, a transgenic line expressing a *skn-1::gfp* transgene was utilized (Fig. 3f). In normal conditions, SKN-1 is present in the nuclei of ASI sensory neurons (Fig. 3g), but it translocates to the nucleus in the intestine in response to oxidative stress from various sources, including the mitochondrial toxin sodium arsenite (5 mM) (Fig. 3f,h). The transgenic worms were simultaneously exposed to 5 mM sodium arsenite and different concentrations (0.1–0.2 mM) of TCS for 30 min. The nuclear localization of SKN-1 was subsequently analysed by fluorescence microscopy. Remarkably, the nuclear localization of SKN-1::GFP in the intestine was suppressed even at 0.1 mM TCS (Fig. 3i–k), which confirmed that TCS inhibited the nuclear localization of SKN-1 and the expression of its target genes (e.g., *gcs-1* and *pmp-3*) (Fig. 3a). We next tested whether constitutively activated SKN-1 could increase resistance to TCS-induced toxicity. For this purpose, we employed a *skn-1(lax120)* gain-of-function (gf) mutant (henceforth called *skn-1(gf)*), which has been shown to activate the expression of genes associated with metabolism, adaptation to starvation, aging, and survival^39^. The *skn-1(gf)* mutant worms were exposed to lethal concentrations (≥0.4 mM) of TCS for 1 hour and the survival rates were compared with those of wild-type control worms. The *skn-1(gf)* mutants, in comparison to wild-type worms, were more resistant to the lethal concentration of TCS (Fig. 3l) and exhibited decreased TCS-induced ROS levels (Fig. 3m). These findings suggest that constitutive SKN-1 activation protects worms against TCS-induced toxicity and oxidative stress.

### TCS inhibits the expression of SKN-1 target, GCS-1

In *C. elegans*, oxidative stress induces the expression of *gcs-1*, a direct downstream target of SKN-1, in the intestines^15^. To determine whether TCS inhibits the expression of *gcs-1* through SKN-1, we used a transgenic worm that expresses a *gcs-1 promoter::gfp* transgene^15^. L1-staged *gcs-1::gfp* transgenic worms were exposed to SA (0.2 mM; non-lethal concentration) and TCS (0, 0.1, and 0.2 mM) for 6 hours at 20 °C. Under normal conditions, GCS-1::GFP was found to be detectable in the pharynx, but its expression was very weak in the intestine^15^. As previously reported^15^, SA exposure dramatically increased GCS-1::GFP expression in the anterior and posterior regions of *C. elegans* intestine (Fig. 3n,q). Notably, TCS exposure decreased the SA-induced GCS-1::GFP expression in the intestine (Fig. 3o–q). GCS-1 synthesizes GSH, which is critical for the detoxification of oxidants and toxins^33^. Moreover, GSH levels are elevated in many types of tumor cells (e.g., human ovarian carcinoma) that show increased resistance to chemotherapeutic agents^40,41^. To test whether GSH supplementation could protect worms against TCS-induced toxicity, wild-type worms were pre-incubated with different concentrations of GSH (0–0.6 mM) for 1 hour at 20 °C, and then exposed to a lethal concentration of TCS (0.4 mM) for an additional 1 hour. Notably, the survival rate was increased by GSH supplementation in a dose-dependent manner (Fig. 3r). These findings suggest that TCS induces cellular toxicity, at least in part, by disrupting SKN-1/GCS-1-mediated oxidative stress response.

### TCS affected hMSC proliferation rate

**Figure 4:**
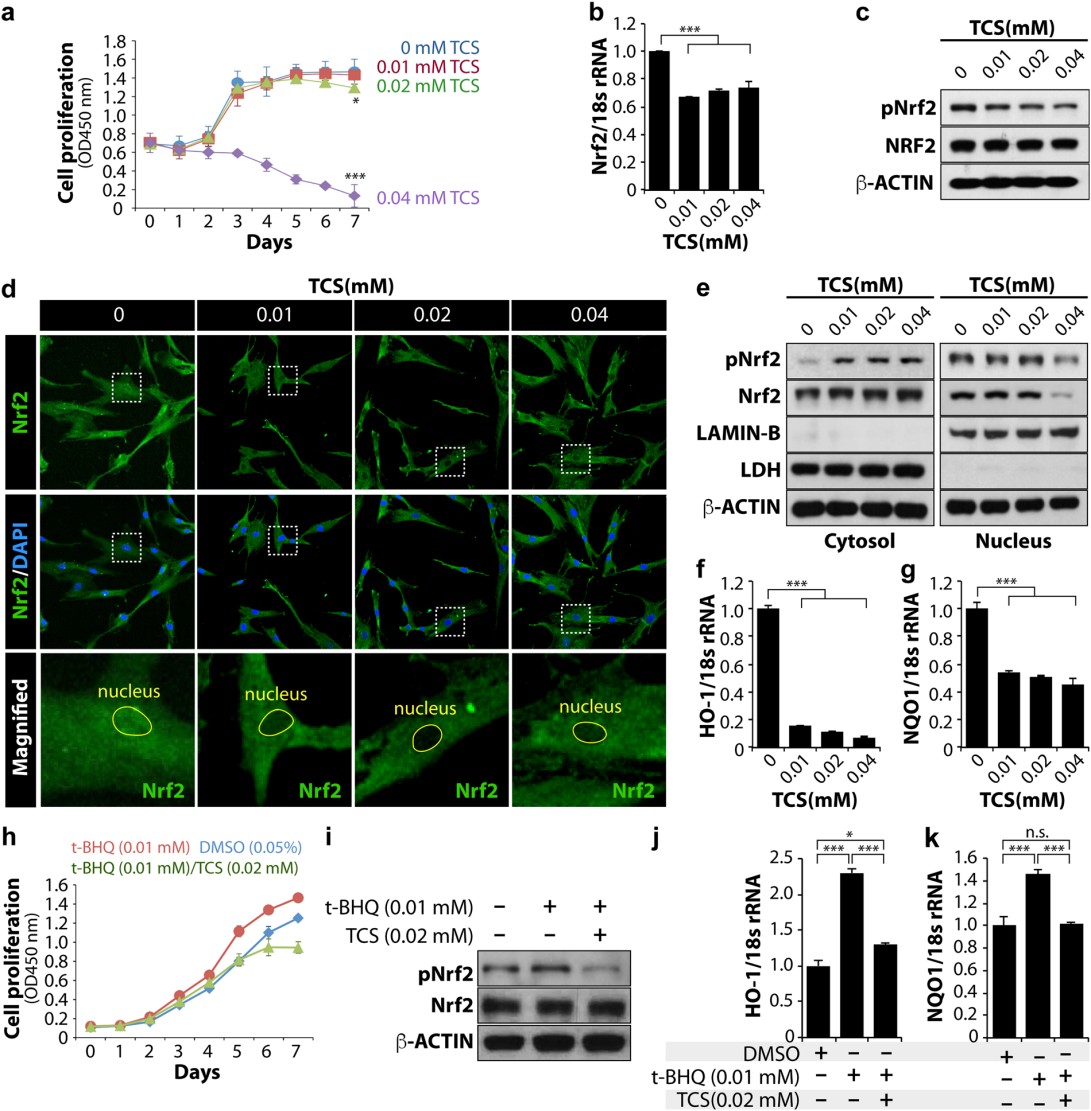
TCS induces cytotoxicity in hMSCs by blocking the nuclear localization of Nrf2 protein. (**a**) Cell proliferation assay; each experiment was performed in triplicate. (**b**) EtOH or TCS-treated MSCs were collected at 24 hours to prepare cell lysates. The levels of *Nrf2* mRNA were analysed by qRT-PCR. (**c**) The levels of total Nrf2 and phosphorylated Nrf2 proteins were determined by a western blot analysis. β-Actin was used as the loading control. (**d**) Immunofluorescence was performed to confirm the nuclear and cytosolic localization of Nrf2 proteins. DAPI was used to stain cell nuclei, and FITC-conjugated secondary antibody was used to visualize Nrf2 protein. The images were obtained using confocal microscopy. (**e**) Cytosolic and nuclear extracts were prepared and fractionated in accordance with the manufacturer’s instructions. The protein levels of total Nrf2 and phosphorylated Nrf2 were analysed by western blot in the EtOH and TCS-treated human cells. The protein level of LDH (lactate dehydrogenase) was used as a loading control for the cytosolic extracts and the protein level of LAMIN-B was used as a loading control for nuclear extracts. (**f**,**g**) *HO-1* and *NQO-1* mRNA levels were analysed by qRT-PCR. *18S rRNA* was used as a loading control. (**h**–**k**) TCS blocks t-BHQ-induced Nrf2 nuclear localization and the expression of its target genes. (**h**) Cell proliferation assay. (**i**) The levels of total Nrf2 and phosphorylated Nrf2 proteins were determined by a western blot analysis. β-Actin was used as the loading control. (**j**,**k**) *HO-1* and *NQO-1* mRNA levels were analysed by qRT-PCR. *18S rRNA* was used as a loading control. Standard deviation bars were calculated from at least three independent experiments. *p* < 0.05(*); *p* < 0.01(**); *p* < 0.001(***); Not statistically significant (n.s.).

Next, we used bone marrow-derived human mesenchymal stem cells (hMSCs) to explore whether the findings observed in worms were conserved in vertebrates. hMSCs have demonstrated great potential in the development of *in vitro* human assays and new target and drug discovery. These stem cells can be readily expanded *in vitro* and can be isolated from a variety of tissue sources. In particular, hMSCs are genetically manipulable to differentiate into adipocytes, chondrocytes, and osteocytes. These features allow for a variety of disease-related drug screening efforts directed towards stem cell self-renewal, proliferation, and differentiation. Using hMSCs, we first assessed the effect of TCS on the proliferation rate. Early passage (EP)-hMSCs were exposed to four different concentrations (0, 0.01, 0.02, and 0.04 mM) of TCS for 7 days and the proliferation rates were measured using an EZ-Cytox kit. No difference in the proliferation rate between control EP-hMSCs and 0.01 and 0.02 mM TCS-treated EP-hMSCs was observed (Fig. 4a). However, the proliferation rate of 0.04 mM TCS-treated EP-hMSCs was dramatically reduced after 2 days (Fig. 4a). These results indicated that TCS affected the proliferation rate of hMSCs in a dose-dependent manner.

### TCS exposure blocked Nrf2 activation in hMSCs

To determine whether TCS could affect the expression of *Nrf2* mRNAs and proteins in hMSCs, EP-hMSCs were treated with 0, 0.01, 0.02, and 0.04 mM TCS for 12 hours. The expression levels of *Nrf2* mRNAs were normalized to that of *18 s rRNA*. The qRT-PCR results showed that TCS slightly reduced the expression of *Nrf2* mRNA at all doses tested (Fig. 4b). We also examined the expression level of Nrf2 protein by western blot using an anti-Nrf2 antibody. TCS exposure only slightly reduced the protein level of Nrf2, whereas the level of phosphorylated (activated) Nrf2 (pNrf2) proteins was significantly decreased by TCS exposure dose-responsively (Fig. 4c). pNrf2 reflects nuclear localization where it activates the expression of its target genes. To visualize the nuclear localization of pNrf2 proteins in hMSCs, we performed immunocytochemical analysis using an anti-Nrf2 antibody. In agreement with the western blot results, only a small amount of nuclear pNrf2 was detected in the TCS-treated hMSCs (Fig. 4d). To compare these phenomena quantitatively, the cytosolic and nuclear fractions of each experimental group were prepared and analysed by western blotting. The level of nuclear pNrf2 protein was decreased in TCS-treated hMSCs, especially at a dose of 0.04 mM TCS (Fig. 4e). In contrast, cytosolic levels of pNrf2 proteins increased in TCS-treated hMSCs (Fig. 4e). We next examined the mRNA levels of *HO-1* (encoding haem oxygenase-1) and *NQO-1* (encoding NAD(P)H quinone dehydrogenase-1) mRNAs, which are known as downstream target genes of Nrf2, by qRT-PCR. Notably, the expression levels of *HO-1* and *NQO-1* were significantly decreased by TCS treatment (Fig. 4f,g). These results suggest that TCS exerted an ability to block the nuclear localization of pNrf2 protein, whereas the levels of the cytosolic Nrf2 proteins were unchanged by TCS treatment.

### TCS exposure inhibited t-BHQ-mediated Nrf2 activation

Under normal conditions, human Nrf2 exists in the cytoplasm and is degraded via ubiquitination triggered by binding to Kelch-like ECH-associated protein 1 (Keap1)^42^. However, under oxidative stress, Nrf2 is phosphorylated at Ser40 by protein kinase C^43^ and at multiple sites by MAP kinases^44^ to translocate to the nucleus by disrupting the complex with Keap1 in the cytoplasm^45,46^. *t*-BHQ (*tert*-butylhydroquinone, antioxidant agent) is an activator of Nrf2. *t*-BHQ promotes Nrf2 stabilization by preventing binding with Keap1^47^, resulting in a higher hMSC proliferation rate. To test whether TCS inhibited t-BHQ-induced hMSC proliferation rate, cells were treated with 0.05% DMSO control, t-BHQ (0.01 mM), and a t-BHQ (0.01 mM)/TCS (0.02 mM) mixture for 7 days, and the proliferation rates were determined. As we previously reported^48^, t-BHQ (0.01 mM) treatment enhanced the proliferation rate compared with the DMSO control (Fig. 4h). However, TCS (0.02 mM) suppressed the t-BHQ-induced proliferation rate after day 3 (Fig. 4h). Next, to test if TCS inhibited t-BHQ-mediated Nrf2 phosphorylation, we treated hMSCs with either t-BHQ (0.01 mM) or t-BHQ (0.01 mM)/TCS (0.02 mM) for 12 hours and measured the expression levels of pNrf2 and total Nrf2 by western blotting. Intriguingly, t-BHQ markedly increased the level of pNrf2, but 0.02 mM TCS decreased the levels of t-BHQ-induced pNrf2 (Fig. 4i). However, no changes in the levels of total Nrf2 proteins were observed in t-BHQ alone or t-BHQ/TCS-treated hMSCs (Fig. 4i). Next, we determined whether TCS inhibited the t-BHQ-induced expression of Nrf2 target genes by analysing t-BHQ- and tBHQ/TCS-treated hMSC extracts by qRT-PCR. The levels of *HO-1* and *NQO-1* transcripts were normalized to the level of *18 s rRNA*. As previously reported^49^, t-BHQ significantly increased the transcript levels of *HO-1* and *NQO1* transcripts, but TCS efficiently suppressed this increase (Fig. 4j,k). These results suggested that TCS inhibited the nuclear localization of pNrf2 and subsequently suppressed the expression of its target genes, which are associated with oxidative stress-mediated defense mechanisms.

## Discussion

**Figure 5:**
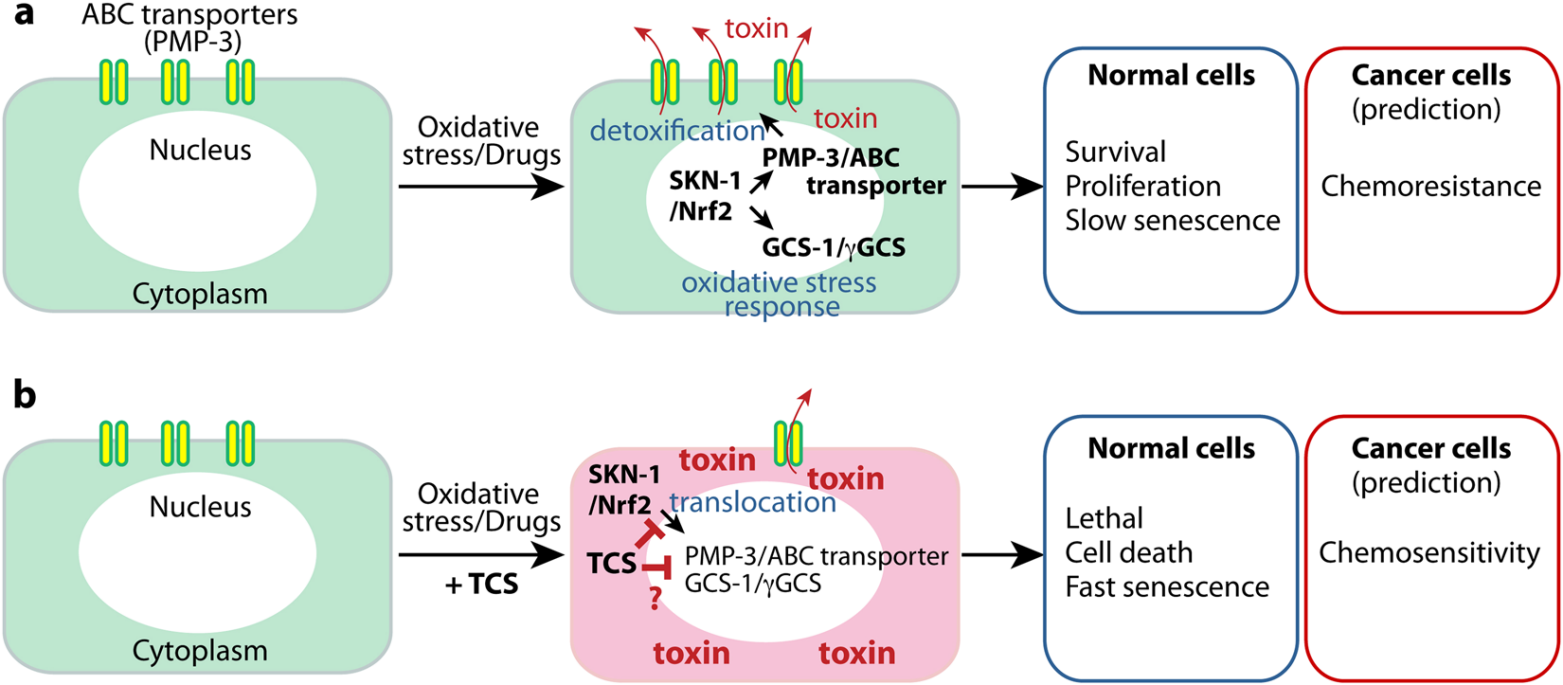
A proposed mechanism of action for TCS in normal and cancer cells. See text for details.

This work demonstrated that TCS induced oxidative stress by the disruption of SKN-1/Nrf2-mediated oxidative stress response, in two powerful model systems, *C. elegans* and hMSCs. Based on our findings, we have suggested a possible action mechanism of TCS in SKN-1/Nrf2-mediated oxidative stress response and detoxification (Fig. 5). Under oxidative stress conditions, pSKN-1/pNrf2 proteins translocate from the cytoplasm to the nucleus and then protect cells or animals from oxidative stress by the activation of target genes (e.g., *gcs-1* and *pmp-3*), which are associated with oxidative stress response and detoxification mechanisms. GCS-1/γGCS plays an important role in the detoxification of oxidants and toxins from cells^33^. The PMP-3/ABC transporter mediates the efflux of molecules, including metabolites and toxic agents (e.g., drug, chemicals). However, TCS inhibited the translocation of pSKN-1/pNrf2 proteins into the nucleus and thereby suppressed the expression of the *gcs-1*/γGCS1 and *pmp-3*/ABC transporter genes. Therefore, we suggest that TCS induces oxidative stress and intracellular accumulation of toxins, at least in part, through disrupting SKN-1/Nrf2-mediated oxidative stress response and detoxification mechanism.

Oxidative stress is defined as an imbalance between the production of reactive oxygen species (ROS) and their elimination by protective mechanisms. This imbalance leads to the oxidative damage of biomolecules and cells within the body. Oxidative stress intertwine with the multiple stages of the cancer process, including initiation, promotion, and progression^50–52^. Moreover, ROS plays a major role in tumor initiation and survival, as induced by a variety of agents in several model systems, including humans. ROS-induced oxidative stress can be reduced by the main cellular defense mechanisms^53^. One of the major regulators is Nrf2, which activates the cellular antioxidant response by inducing the transcription of its target genes. Nrf2 target genes protect cells from the effects of xenobiotics and oxidative stress^54^ and inhibit carcinogenesis^55^. Therefore, although Nrf2 has been generally considered as a tumor suppressor, it can also protect cancer cells from oxidative stress and chemotherapeutic agents (Fig. 5). Recent studies have revealed that Nrf2 pathway activation favoured tumor survival with an increased chemoresistance to standard chemotherapy^54^. Although whether the activation or inhibition of Nrf2 is useful for the prevention or treatment of cancer remains a highly controversial issue, identification of Nrf2 inhibitors will be of value to the development of cancer therapeutic advances. For instance, brusatol was previously identified as a chemical inhibitor of the Nrf2 pathway^56^. Brusatol was able to enhance the efficacy of chemotherapy through the inhibition of Nrf2-mediated defense mechanisms^56^. Mechanistically, brusatol selectively decreased Nrf2 protein levels through enhanced ubiquitination and degradation^56^. Importantly, brusatol sensitized cancer cells and xenografts to chemotherapeutic drugs, such as cisplatin, in an Nrf2-dependent manner^56^. Our studies have also identified TCS as a potential chemical inhibitor of the Nrf2 pathway. Although the detailed mechanism by which TCS inhibited the nuclear localization of pNrf2 proteins was not addressed in this study, future research not only will address this aspect, but will also greatly contribute to our understanding of Nrf2-mediated defense mechanisms and chemoresistance.

Various environmental stresses can induce the excessive production of ROS, which triggers cellular senescence and abnormal differentiation in hMSCs^57^. The results in *C. elegans* and hMSCs indicated that TCS may induce cellular toxicity by the disruption of SKN-1/Nrf2-mediated oxidative stress response. Specifically, TCS inhibited the nuclear localization of Nrf2 in EP-hMSCs, and consequently, the expression of its downstream target genes (e.g., *HO-1* and *NQO-1*). These results suggest that TCS may promote the senescence of EP-hMSCs. It was recently reported that Nrf2 activation by t-BHQ promotes stem cell capacity in late passage (LP)-hMSCs through Sirt1 expression^48^. After stimulation by t-BHQ, Nrf2 proteins are released from Keap1 (Kelch-like ECH-Associated Protein 1) and translocate into the nucleus^58^. t-BHQ treatment in LP-MSCs promotes Nrf2 phosphorylation, which activates the expression of target genes (*HO-1* and *NQO1*) and *Sirt1* by similar levels to those observed in EP-hMSCs. Intriguingly, t-BHQ-induced activation of Nrf2 in LP-hMSCs promoted stem cell capacity of hMSCs with increased proliferation and differentiation abilities into osteogenic and adipogenic lineages. These results suggest that active Nrf2 may mediate Sirt1 to regain stem cell capacity in LP-hMSCs. As TCS affected Nrf2 nuclear localization, we proposed that TCS may also affect stem cell capacity during aging. Altogether, since Nrf2 pathways are involved in multiple physiological processes during development and in cancer cell chemoresistance, our findings provide a paradigm that may facilitate pharmacological approaches to therapeutic targeting and disease modelling.

## Materials and Methods

### Chemicals

Triclosan (5-Chloro-2-(2,4-dichlorophenoxy) phenol, TCS), DEET (*N*,*N*-diethyl-*meta*-toluamide), and methylparaben (Methyl 4-hydroxybenzoate, MP) as shown in Fig. 1a were purchased from Sigma Aldrich (MO, USA). All other solvents and chemicals used in the study were of analytical grade. See Supplementary Table 7 for chemical information.

### Preparation of chemical stock solution and chemical treatment

TCS, DEET, and MP powders were dissolved in ethanol as a solvent. To investigate the toxicity of selected chemicals, all stock solutions (100 mM) were diluted to final concentrations of 0.1, 0.2, and 0.4 mM with the sterilized NGM (Nematode Growth Media: 3 g NaCl, 2.5 g Pepton, 17 g Agar, 1 ml 1 M CaCl_2_, 1 ml 1 M MgSO_4_, 25 ml 1 M KPO_4_ (pH 6.0), 1 ml Cholesterol (5 mg/1 ml), H_2_O to 1 litre) media before plating (see Fig. 1b) or M9 buffer (3 g KH_2_PO_4_, 6 g Na_2_HPO_4_, 5 g NaCl, 1 ml 1 M MgSO_4_, H_2_O to 1 litre) (see Fig. 1j). For long-term TCS exposure in NGM plates (see Fig. 1b), isolated wild-type embryos were plated on NGM plates containing the selected chemicals for 36 hours at 20 °C. The survival rate was determined every 12 hours by scoring live and dead worms under a dissecting microscope. For short-term TCS exposure in M9 liquid buffer (see Fig. 1j), synchronized L1 stage larvae were incubated in M9 buffer containing the selected chemicals for 1 hour at 20 °C. Following treatment, worms were washed with fresh M9 buffer to remove remaining traces and were transferred to the NGM plates seeded with *E. coli* OP50. TCS solution (0 mM to 0.4 mM) and mixtures of TCS, DEET, and MP (0.2 mM) of each were prepared by adding appropriate quantities of solutes to a volumetric flask and filling to the mark with ethanol.

### *C. elegans* strains and culture conditions

Wild-type N2, other mutants and transgenic worms were obtained from the Caenorhabditis Genetics Center (CGC) (see Supplementary Table 1). These strains were cultured at 20 °C under standard growth conditions on an NGM agar plates seeded with *E. coli*. OP50. For toxicology studies, the worms were synchronized using a bleaching solution (0.5 M NaOH and 1.2% NaClO). Then, the obtained embryos or L1 stage larvae were exposed to the solution consisting of either only TCS or a combination of TCS with DEET and MP for 1 hour at 20 °C, and the effects of the chemicals on survival rate, lifespan, and gene expression were measured.

### Survival assay

The survival assays were performed using wild-type(N2) and three *mev-1(kn1)*, *gcs-1(ok436)*, *and pmp-3(ok1087)* mutants (see Supplementary Table 1). After 1 hour of chemical exposure, synchronized L1 stage larvae were washed three times with fresh M9 buffer and transferred to the NGM plates with *E. coli* OP50. Their viability was measured immediately or every 12 hour under a dissecting microscope. Test worms were considered dead when they failed to respond to prodding with the tip of a platinum wire. Standard deviation bars were calculated from at least three independent experiments.

### Lifespan assay

The lifespan assays were performed using wild-type(N2) and *mev-1(kn1)* mutants. Synchronized L1 stage larvae were transferred to the NGM plate in the absence or presence of TCS (0.02 mM and 0.04 mM). The worms were transferred to fresh NGM plate every 2 days and dead worms were counted daily. Standard deviation bars were calculated from three independent experiments.

### Measurement of intracellular ROS (Reactive Oxygen Species)

Intracellular ROS in the worms was measured using molecular probe 2′,7′-dichlorodihydrofluoroscein diacetate (H_2_DCF-DA). Wild-type(N2) or mutant worms were exposed to either EtOH or TCS for the measurement of intracellular ROS. Chemical-treated L1-arrested larvae were then transferred into the 96-well plate containing 50 μL of M9 buffer. Immediately 50 μL of 50 μM H_2_DCF-DA was added to each well. The basal fluorescence was quantified using a microplate fluorescence reader at an excitation wavelength of 485 nm and an emission wavelength of 535 nm. Plates were read every 30 minutes for 5 hours. Three replicate experiments were conducted.

### Gene expression analysis

After the exposure of L1 stage larva to 0.02 mM and 0.04 mM of TCS, about 2,000 L1 stage larvae were transferred to fresh NGM plates seeded with *E.coli* OP50. These worms were then incubated for 2 days at 20 °C before they were harvested in 1.5 ml microtube. Total RNA was extracted from the worms using *mir*Vana^TM^ miRNA Isolation Kit (Life Technologies). Quality and purity of total RNA was quantified by a Nanodrop ND-1000 (Nanodrop Technologies, Wilmington, DE, USA). For reverse transcription (RT), 650 ng of total RNA from control and each treatment sample were used. RNA was transcribed to single-stranded cDNA using the reverse primer Poly(dT). RT-PCR was conducted in 250 μL microcentrifuge tubes and reacted in a 15 μL solution. This solution consists of precalculated amounts of DNase/RNase-free water, 1,000 ng of total RNA, 0.19 μL RNase inhibitor (20 U/μL), 0.15 μL 100 mM dNTPs, 10× RT buffer, 1 μL MultiScribe^TM^ reverse transcriptase (50 U/μL), and 2 μL of Poly(dT) primer mix. The reactions were performed using an Eppendorf Mastercycler® Pro PCR machine. Quantitative real-time PCR (qRT-PCR) reactions were performed in 10 μL solutions, consisting of 2 μL DNase/RNase free water, 5 μL SYBR green PCR master mix, 1 μL RT PCR product (diluted with 85 μL of DNase/RNase free water), and 2 μL of primer mix. The reactions were performed with a VIIA^TM^ 7 Real-Time PCR system using programmed temperature. The programmed temperature evaluation starts with an initial polymerase activation stage for 10 minutes at 95 °C, followed by denaturation for 15 seconds at 95 °C, and an annealing/extension step for 60 seconds at 60 °C. The latter two steps were repeated for 40 cycles. Four biological replicates were performed per treatment group with each having three technical replicates. In qRT-PCR, the expression was normalized to *tba-1*, which encodes *α-tubulin*, and *18S rRNA* ^59–61^.

### GSH rescue assay

Wild-type L1 worms were pretreated with GSH (0–0.6 mM) in M9 buffer for 1 hour and then were treated with 0.4 mM TCS for additional 1 hour. Worms were washed three times with M9 buffer and then transferred to NGM agar plates. Their viability was measured under a dissecting microscope. Standard deviation bars were calculated from at least three independent experiments.

### SKN-1::GFP expression analysis

To analyze the effect of TCS on the localization of SKN-1::GFP in response to oxidative stress, *skn-1::gfp* transgenic worms were exposed to 5 mM sodium arsenite for 30 minutes at 20 °C in the absence or the presence of TCS (0.1–1 mM), then covered with a glass slip and examined by fluorescence microscopy. These worms were scored for the presence of SKN-1::GFP in intestinal nuclei 5 minutes later under a fluorescence microscope.

### *gcs-1::gfp* expressional analysis

To analyze the induction of GCS-1::GFP expression in the absence or presence of TCS under oxidative stress condition, LD1171 (*ldIs3* [*gcs-1p::GFP* + *rol-6(su1006)*]) worms were exposed to 0.2 mM sodium arsenite for 6 hours at 20 °C in the absence or presence of TCS (0–0.2 mM). The GCS-1::GFP expression levels were examined under a fluorescence microscope.

### Mesenchymal stem cell (MSC) preparation and cultivation

Methods for MSC isolation and cultivation were previously described^62^. In brief, bone marrow aspirates were collected from the posterior iliac crests of three adult donors, with approval from the Institutional Review Board of Yonsei University College of Medicine. The cells were cultured according to previously published protocols^48^, and their characteristics were confirmed using flow cytometry^63^. MSCs at passage 1 to passage 3 were mainly used in this experiment. TCS was dissolved in ethanol, which also served as a control, to prepare testing solutions of 0.01 mM, 0.02 mM, and 0.04 mM.

### Cytotoxicity assay (Ez-Cytox)

Cell cytotoxicity for TCS was examined using an EZ-Cytox Kit (Daeil Lab Service, Seoul, Korea). TCS-treated MSCs were seeded in 12-well cell culture plates 1 × 10^4^ cells per well. TCS-treated MSCs were maintained in growth media for 7 days, and the growth media were changed every 2 days. In brief, the cells were washed by phosphate-buffered saline (PBS), and then 1 mL growth media containing 20 μl of EZ-Cytox (tetrazolium salts) solution was added to each well and incubated at 37 °C for 3 hours. Then, the conditioned media were transferred into 96-well plates, and the absorbance was measured at 450 nm using spectrophotometry. All sample were tested in triplicate (n = 3).

### Quantitative real-time polymerase-chain reaction (qPCR)

RNA was isolated using an RNeasy kit (Qiagen, Valencia, CA, USA). The isolated RNAs were then reverse-transcribed using an Omniscript kit (Qiagen). Primer sets were validated and purchased from Bioneer (Daejeon, South Korea; http://www.bioneer.co.kr/). The primers used were as follows: *Nrf2* [P164742], *HO-1* [P133045], and *NQO-1* [P113225]. *18S rRNA* was used as an internal control and the sequence was as follows: 5′-acacggacaggattgacagattg-3′ (sense, NR_003286.2) and 5′-gccagagtctcgttcgttatcg-3′ (antisense). Mean cycle threshold values from triplicate (n = 3) measurements were used to calculate gene expression, with normalization to *18S rRNA* levels.

### Cytosol and nucleus extraction

Ethanol or TCS-treated MSCs were collected by trypsinization, and then nuclear and cytosolic fractionation was conducted using the NE-PER Nuclear and Cytoplasmic Extraction Reagents Kit (Thermo Fisher Scientific, Rockford, IL, USA) according to the manufacturer’s instructions. The nuclear and cytosolic proteins were analyzed by western blot analysis.

### Western blot

Ethanol- or TCS-treated hMSCs were lysed in passive lysis buffer (Promega, Madison, WI, USA) after 12 hours of TCS treatment. The Bio-Rad protein assay (Bio-Rad Laboratories, Hercules, CA, USA) were used to determine protein concentrations, and 10 μg of proteins were analyzed by electrophoresis on a 10% sodium dodecyl sulfate-polyacrylamide gel (Sigma). 5% skim milk (BD, Sparks, MD, USA) was used to block the transferred membranes, and the membranes were incubated for overnight with antibodies against Nrf2 (Santa Cruz Biotechnology, Santa Cruz, CA, USA), phosphorylated-Nrf2 (Abcam, Cambridge, UK), LAMIN-B (Santa Cruz Biotechnology), and LDH (Santa Cruz Biotechnology). The membranes were further probed with an antibody against β-ACTIN (Santa Cruz Biotechnology), which served as a loading control. See Supplementary Table 2 for the information of antibodies, used for this study.

### Immunocytochemistry

Ethanol- or TCS-treated hMSCs were seeded at 2,000 cells per square centimeter on four-well glass chamber slides (Nalge Nunc International, Rochester, NY, USA), and the cells were incubated in a 5% CO_2_ incubator at 37 °C. The cells were fixed with 4% paraformaldehyde (Sigma) for 30 minutes, and then permeabilized with 1% Triton X-100 for 10 minutes followed by blocking for 1 hour with 5% bovine serum albumin (BSA) in PBS. The cells were incubated with 1:100 dilution of antibodies against Nrf2 (Santa Cruz Biotechnology) for 4 hours at room temperature, and then incubated fluorescein isothiocyanate (FITC)-conjugated secondary antibodies (Santa Cruz Biotechnology) at a 1:5,000 dilution in 1% BSA-containing PBS for 1 hour at room temperature in the dark. The nuclei were stained with 4,6-diamidino-2-phenyindole (DAPI) (Sigma) and then examined using a Zeiss LSM700 scanning laser confocal microscope (Zen 2011; Carl Zeiss MicroImaging GHBH, Jena,Germany).

### Data analysis

The data from the survival rate analysis and lifespan assay were plotted using Microsoft Excel software and statistical significance was analyzed by log-rank test. Other data were presented as mean ± standard deviation or standard error of the mean depends on the experiments as indicated. Statistical significance of differences between the control and treated groups were analyzed by one-way analysis of variance (ANOVA). The error bars reflect respective standard deviation values.

## Electronic supplementary material

Supplementary Info
