# Supplementary material for: Triclosan Disrupts SKN-1/Nrf2-Mediated Oxidative Stress Response in C. elegans and Human Mesenchymal Stem Cells
Source: Sci Rep. 2017 Oct 3;7:12592. doi: 10.1038/s41598-017-12719-3 (PMC5626723; doi:10.1038/s41598-017-12719-3)
Supplement: Supplementary file 1 — Supplementary Info [file 41598_2017_12719_MOESM1_ESM.pdf]

Supplementary Information for

Triclosan Disrupts SKN-1/Nrf2-Mediated Oxidative Stress Response in *C. elegans* and Human Mesenchymal Stem Cells

Dong Suk Yoon[1,2,10], Yoorim Choi[2,3,10], Dong Seok Cha[1,4,10], Peng Zhang[5], Seong Mi Choi[2,3], Mohammad Abdulmohsen Alfhili[1,6], Joseph Ryan Polli[7], DeQwon Pendergrass[1,7], Faten A. Taki[7], Brahmam Kapalavavi[8], Xiaoping Pan[7], Baohong Zhang[7], T. Keith Blackwell[5], Jin Woo Lee[2,3,*], and Myon-Hee Lee[1,9,*]

[1]Department of Internal Medicine, Brody School of Medicine at East Carolina University, Greenville, NC 27834, USA. [2]Department of Orthopaedic Surgery, Yonsei University College of Medicine, Seoul 120-752, South Korea. [3]Brain Korea 21 PLUS Project for Medical Sciences, Yonsei University College of Medicine, Seoul 120-752, South Korea. [4]Present address: Department of Oriental Pharmacy, College of Pharmacy, Woosuk University, Jeonbuk 565-701, Republic of Korea. [5]Research Division, Joslin Diabetes Center, Boston, MA 02215, USA; Department of Genetics and Harvard Stem Cell Institute, Harvard Medical School, Boston, MA 02115, USA. [6]Department of Clinical Laboratory Sciences, College of Applied Medical Sciences, King Saud University, Riyadh, Saudi Arabia 11433. [7]Department of Biology, East Carolina University, Greenville, NC 27858, USA. [8]Department of Chemistry, East Carolina University, Greenville, NC 27858, USA. [9]Lineberger Comprehensive Cancer Center, University of North Carolina-Chapel Hill, Chapel Hill, NC 27599, USA. [10]These authors equally contributed to this work. *Correspondence and requests for materials should be addressed to J.W.L (email: ljwos@yuhs.ac.kr) or M.H.L (email: leemy@ecu.edu).

**Supplementary Tables**	
Table 1	*C. elegans* strains and transgenic lines
Table 2	Antibodies that were used for this study
Table 3	Statistical analysis of lifespan (see Fig. 2e-h)
Table 4	Description of the 21 tested genes
Table 5	Primer information for Fig. 3a
Table 6	Primer information for Fig. 4
Table 7	Chemical information
**Supplementary Figures**	
Figure 1	The effects of TCS on wild-type worm survival in M9 liquid buffer (Supporting Information for Fig. 1k)
Figure 2	TCS toxicity between L1 larvae and adult worms
Figure 3	The localization of Nrf2 proteins in EP-hMSCs (Supporting information for Fig. 4d).
Figure 4	Original full images of immunoblots (Supporting information for Fig. 4)

Supplementary Table 1. *C. elegans* strains and transgenic lines

Strain	Genotype	Source
N2	wild-type (N2)	CGC
RB1108	*pmp-3(ok1087)*	CGC
VC337	*gcs-1(ok436)/mIn [mIs14 dpy-10(e128)]*	CGC
TK22	*mev-1(kn1)*	CGC
LD007	*ldEx9 [skn-1(operon)::GFP + rol-6(su1006)]*	Dr. Blackwell
SPC167	*dvIs19 III; skn-1(lax188) IV*	CGC
LD1171	*ldIs3 [gcs-1p::GFP + rol-6(su1006)]*	CGC

Supplementary Table 2. Antibodies that were used for this study.

Antibody	Catalog #	Dilution	Source
Nrf2	sc-722	1:5000 (western blot)<br>1:200 (Immunofluorescence)	Santa Cruz
FITC-conjugated secondary antibody	sc-2012	1:5000 (Immunofluorescence)	Santa Cruz
pNrf2 (S40)	ab76026	1:1000	Abcam
LAMIN-B	sc-6216	1:1000	Santa Cruz
LDH	sc-33781	1:3000	Santa Cruz
β-ACTIN	sc-47778	1:10000	Santa Cruz

Supplementary Table 3. Statistical analysis of lifespan (see Fig. 2e-h). The lifespan (days) of wild-type (N2) and *mev-1(kn1)* mutant worms was determined by the log-rank (Mantel-Cox) test.

Strain	°C	TCS (mM)	Mean Lifespan (days)	Log-Rank Test
wild-type	20	0	14.32 ± 0.40	
	20	0.01	13.54 ± 0.34	p=0.109
	20	0.02	12.64 ± 0.28	p<0.001
	20	0.04	12.12 ± 0.17	p<0.001

Strain	°C	TCS (mM)	Mean Lifespan (days)	Log-Rank Test
mev-1(kn1)	20	0	10.84 ± 0.48	
	20	0.01	9.74 ± 0.44	p=0.139
	20	0.02	8.37 ± 0.46	p=0.001
	20	0.04	8.42 ± 0.40	p<0.001

Supplementary Table 4. Description of the 21 tested genes

Gene	Locus tag	Gene description	Function	Ref.
age-1	B0334.8	AGEing alteration	Phosphoinositide 3-kinase p110 catalytic subunit	8
akt-1	C12D8.10	AKT kinase family	Serine/threonine kinase Akt/PKB	9
ama-1	F36A4.7	AMAnitin resistant	Large subunit of RNA Pol II	10, 11
cep-1	F52B5.5	*C. elegans* p53-like protein	p53 tumor suppressor	12
dct-1	C14F5.1	DAF-16/FOXO Controlled, Germline tumor affecting	BNIP3 ortholog	13, 14
dpl-1	T23G7.1	Vertebrate transcription factor DP-Like	DP ortholog	15, 16
egl-1	F23B12.9	EGg Laying defective	BH3-only domain	17
egl-19	C48A7.1	EGg Laying defective	α1-subunit of Ca^{2+} channel	18
gcs-1	F37B12.2	Gamma GlutamylCysteine Synthetase	Gamma-glutaminecysteine synthetase heavy chain	19
lev-8	C35C5.5	LEVamisole resistant	α-subunit nAChR	20
lin-35	C32F10.2	Abnormal cell LINeage	Retinoblastoma (Rb) ortholog	15
lin-39	C07H6.7	Abnormal cell LINeage	Homeodomain protein homolog of Deformed and Sex combs	21
old-1	C08H9.5	Overexpression Longevity Determinant	RTK	22, 23
pmp-3	C54G10.3	Peroxisomal Membrane Protein related	Acyl-coa transporter, human ABCD4 ortholog	24
rbd-1	T23F6.4	Rna Binding Domain	Mrd1p (S. cerevisiae) ortholog	25
sdhb-1	F42A8.2	Succinate DeHydrogenase Complex subunit B	Succinate dehydrogenase subunit B ortholog	26
skn-1	T19E7.2	SKiNhead	bZip transcription factor (Nrf ortholog)	19, 27
sir-2.1	R11A8.4	Yeast SIR related	Aging and lifespan	28, 29
sod-1	C15F1.7	SuperOxide Dismutase	Copper/zinc superoxide dismutase	30
sod-2	F10D11.1	SuperOxide Dismutase	Iron/manganese superoxide dismutase	31
sod-3	C08A9.1	SuperOxide Dismutase	Iron/manganese superoxide dismutase	31

Supplementary Table 5. Primer information for Fig. 3a.

Gene	Locus Tag	Forward Primer (5'→3')	Reverse Primer (5'→3')	Size (bp)
age-1	B0334.8	GGCAGCCGCGTTGACTTTGC	TGACCGGGCTCAGCTGCTCAT	95
akt-1	C12D8.10	AAGCCTAAGGAAGGACAACC	TCCAACGCTGACGAACTTCTGC	178
ama-1	F36A4.7	CGGATGGAGGAGCATCGCCG	CAGCGGCTGGGGAAGTTGGC	204
cep-1	F52B5.5	AGGGCACGATTCAGTGTTG	TTCATCGCTTCCTGGATGC	171
dct-1	C14F5.1	TGACCGAGAGTACACCTGGTATGTC	ATCGACGCTACTGCACAAACTGG	80
dpl-1	T23G7.1	TACTCTACTGTACCTCCAGATCG	TCTCTGTTGCACTTGATGTGG	186
egl-1	F23B12.9	TCTCAGGACTTCTCCTCGTG	GTCCAGAAGACGATGGAAGA	195
egl-19	C48A7.1	GACGGCCCCAGGTATCGGGA	ACAATCCGAGCGTCGGCGTA	98
gcs-1	F37B12.2	CGAAGAGCAGGTGAATGCGATGC	GCAAGCGATGAGACCTCCGTAAGG	118
lev-8	C35C5.5	CGGCAAGATGATGGCGTGGA	GCGCGATGAAGTGGCACTGAG	105
lin-35	C32F10.2	TGATGATCTACGAGACGAAC	AGATCTTGAAGTCGACGAGC	117
lin-39	C07H6.7	GCGTCTGCTGCACTTTCTGCTC	TGAGGACCTCCCAGTCCTTGAC	104
old-1	C08H9.5	TCCGGGCAAACAGATCAAGAGGT	AGGGAGTTGGTTCTGGTGGAACT	105
pmp-3	C54G10.3	TGGCCGGATGATGGTGTCGC	ACGAACAATGCCAAAGGCCAGC	190
rbd-1	T23F6.4	GGTCAGATTTCCGATGCGTCGCT	ACTTGCTCCAGGCTCTCGGC	199
sdhb-1	F42A8.2	TGGCTGAACGTGATCGTCTTGATGG	TCCACCAGTAGGATGGGCATGACG	84
skn-1	T19E7.2	TTCGGAGATGTCATTAAGCG	TGCGTCTTTGACGGCAAGTGCG	137
sir-2.1	R11A8.4	TACTGAGATGCTCCATGAC	AGCAAGACGAACCACACGAAC	155
sod-1	C15F1.7	CGGACAAGACGACCTCGGCG	GCAATGACACCGCAGGCAGC	96
sod-2	F10D11.1	AGCCAGCTCTCAAGTTCAATGGAGG	AAGTCGCTCTTAATTGCGGTGAGC	118
sod-3	C08A9.1	CCAACCAGCGCTGAAATTCAATGGT	AGTCGCGCTTAATAGTGTCCATCAG	119

Supplementary Table 6. Primer information for Fig. 4

Gene	Primer No	Forward Primer (5'→3')	Reverse Primer (5'→3')	Size (bp)
Nrf2	P164742	TCAGGTAGCCCCTGTTGATTT	GTTTGGCTTCTGGACTTGGAA	149
HO1	P133045	TGCTCAAAAAGATTGCCCAGA	TTCTATCACCCTCTGCCTGAC	167
NQO1	P113225	CCCTGCGAACTTTCAGTATCC	CTTTCAGAATGGCAGGGACTC	158

Primer sets were validated and purchased from Bioneer (Daejon, South Korea: http://www.bioneer.co.kr/)

Supplementary Table 7. Chemical information

Chemical	Formula	Mole. Weight	Catalog #	Source
Triclosan	$C_{12}H_7Cl_3O_2$ 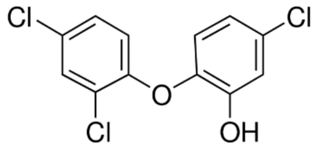	289.54	93453-100MG	Sigma-Aldrich
N,*N*-Diethyl-3-methylbenzamide (DEET)	$C_{12}H_{17}NO$ 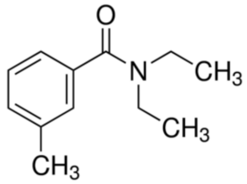	191.27	36542-250MG	Sigma-Aldrich
Methylparaben	$HOC_6H_4CO_2CH_3$ 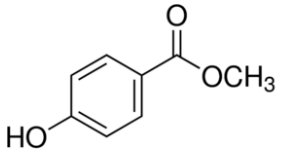	152.15	47889	Sigma-Aldrich
L-Glutathione reduced (GSH)	$H_2NCH(CO_2H)CH_2CH_2CONHCH(CH_2SH)CONHCH_2CO_2H$ 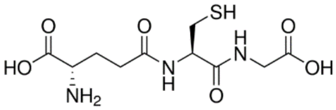	307.32	G4251	Sigma-Aldrich
Sodium Arsenite	$NaAsO_2$ 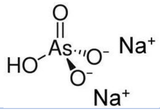	129.91	S7400	Sigma-Aldrich
tert-Butylhydroquinone (t-BHQ)	$(CH_3)_3CC_6H_3$-1,4-$(OH)_2$ 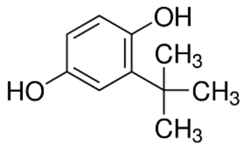	166.22	112941-100G	Sigma-Aldrich

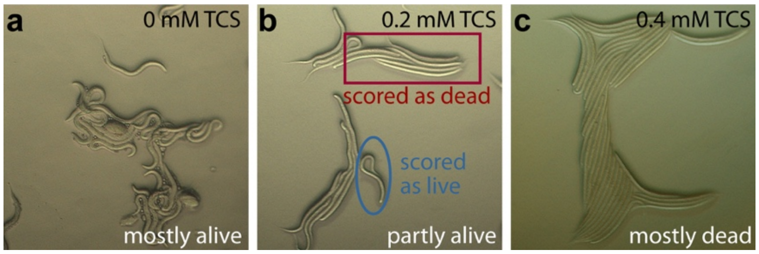

Supplementary Figure 1. The effects of TCS on wild-type worm survival in M9 liquid buffer (Supporting Information for Fig. 1k). (a-c) DIC pictures of wild-type worms treated with 0.4% EtOH control (0 mM TCS), 0.2 mM TCS, or 0.4 mM TCS. The dead worms appear rod-like in shape (see a red box). The live worms move in a liquid solution (see a blue circle).

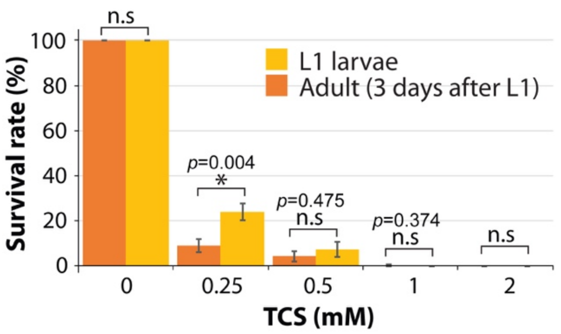

Supplementary Figure 2. TCS toxicity between L1 larvae and adult worms. Synchronized L1 larvae and adult worms (3 days after L1) were exposed to TCS (0-0.4 mM) for 1 h at 20°C and the survival rates were scored as depicted in Fig. 1j.

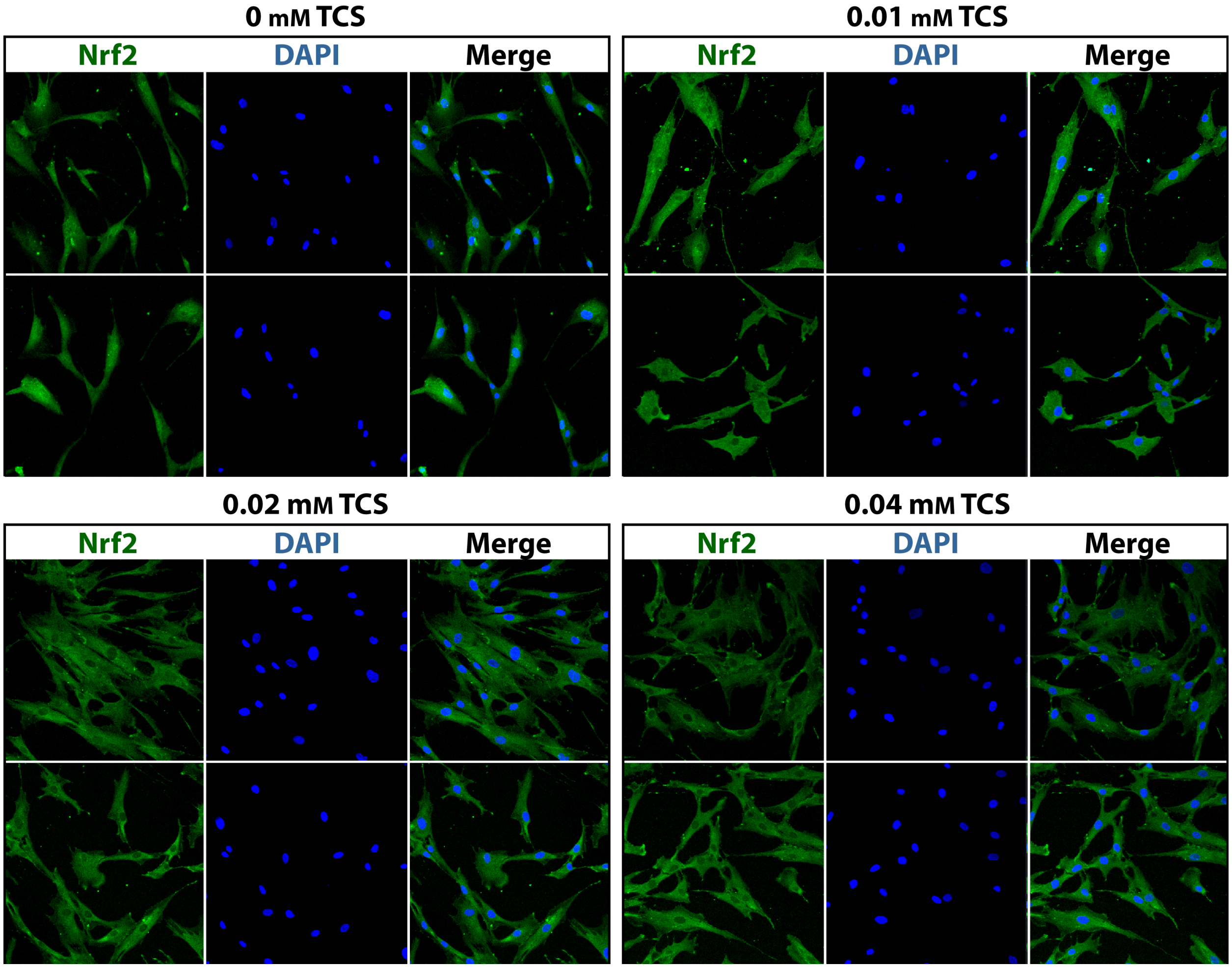

Supplementary Figure 3. The localization of Nrf2 proteins in EP-hMSCs (Supporting information for Fig. 4d). The nuclear and cytosolic localization of Nrf2 proteins were visualized by immunofluorescence.

Supplementary Figure 4. Original full images of immunoblots (Supporting information for Fig. 4)

Figure 3c (pNrf2)

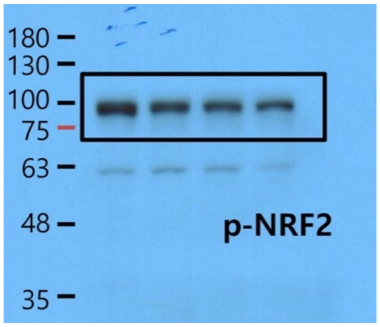

Figure 3c (Nrf2 and β-ACTIN)

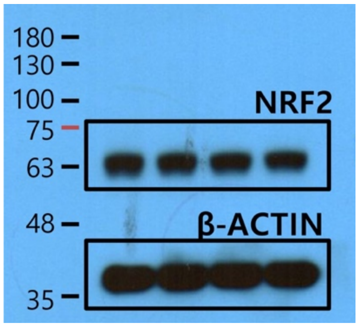

Figure 3e (pNrf2): Cytosol

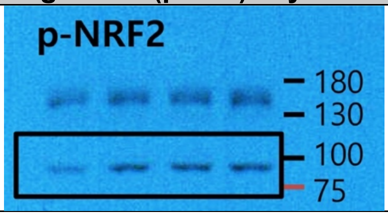

Figure 3e (pNrf2): Nucleus

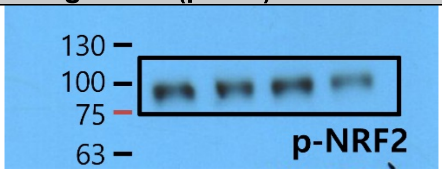

Figure 3e (Nrf2): Cytosol

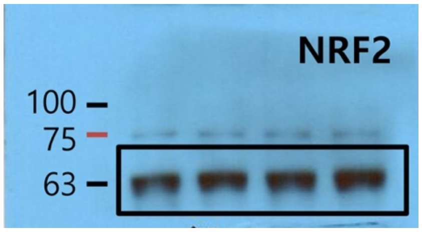

Figure 3e (Nrf2): Nucleus

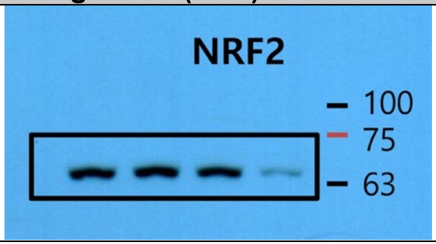

Figure 3e (LAMIN-B): Cytosol

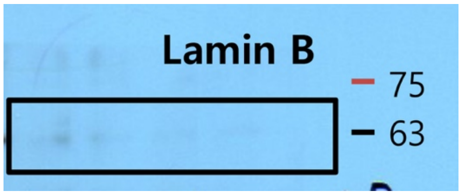

Figure 3e (LAMIN-B): Nucleus

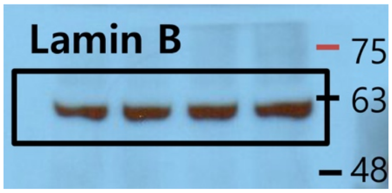

Figure 3e (LDH): Cytosol	Figure 3e (LDH): Nucleus
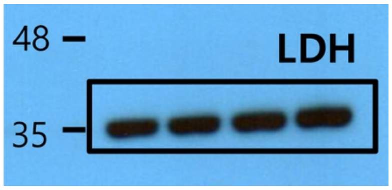	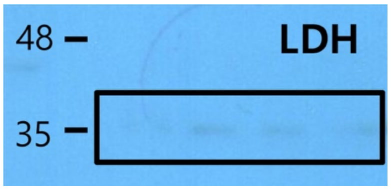

Figure 3e (β-ACTIN): Cytosol	Figure 3e (β-ACTIN): Nucleus
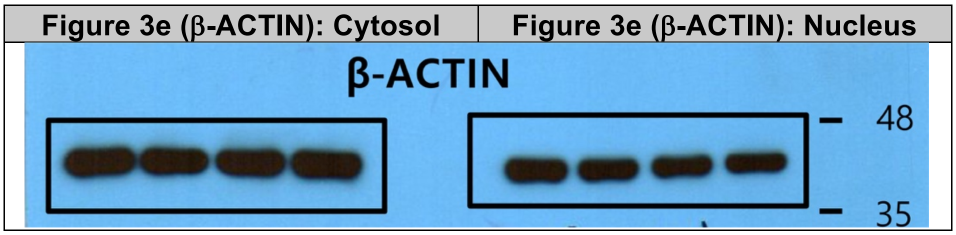	

Figure 3i (pNrf2)	Figure 3i (Nrf2)	Figure 3i (β-ACTIN)
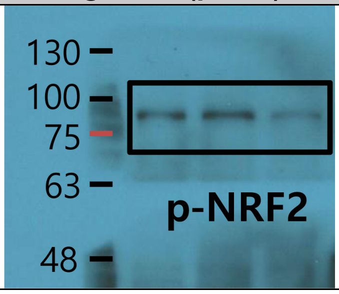	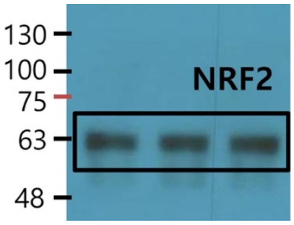	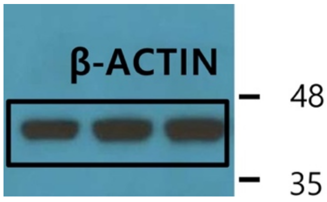

References for Supplementary Information

1. Wu H, Taki FA, Zhang Y, Dobbins DL, Pan X. Evaluation and identification of reliable reference genes for toxicological study in Caenorhabditis elegans. *Molecular biology reports*, (2014).

2. Barnes VM*, et al.* Triclosan blocks MMP-13 expression in hormone-stimulated osteoblasts. *Journal of periodontology* **84**, 1683-1689 (2013).

3. Bedran TB, Grignon L, Spolidorio DP, Grenier D. Subinhibitory concentrations of triclosan promote Streptococcus mutans biofilm formation and adherence to oral epithelial cells. *PLoS One* **9**, e89059 (2014).

4. Cha DS, Datla US, Hollis SE, Kimble J, Lee MH. The Ras-ERK MAPK regulatory network controls dedifferentiation in Caenorhabditis elegans germline. *Biochimica et biophysica acta* **1823**, 1847-1855 (2012).

5. Yoon DS, Kim YH, Jung HS, Paik S, Lee JW. Importance of Sox2 in maintenance of cell proliferation and multipotency of mesenchymal stem cells in low-density culture. *Cell proliferation* **44**, 428-440 (2011).

6. Yoon DS, Choi Y, Lee JW. Cellular localization of NRF2 determines the self-renewal and osteogenic differentiation potential of human MSCs via the P53-SIRT1 axis. *Cell death & disease* **7**, e2093 (2016).

7. Yoon DS*, et al.* Interleukin-6 induces the lineage commitment of bone marrow-derived mesenchymal multipotent cells through down-regulation of Sox2 by osteogenic transcription factors. *FASEB journal : official publication of the Federation of American Societies for Experimental Biology* **28**, 3273-3286 (2014).

8. Morris JZ, Tissenbaum HA, Ruvkun G. A phosphatidylinositol-3-OH kinase family member regulating longevity and diapause in Caenorhabditis elegans. *Nature* **382**, 536-539 (1996).

9. Paradis S, Ruvkun G. Caenorhabditis elegans Akt/PKB transduces insulin receptor-like signals from AGE-1 PI3 kinase to the DAF-16 transcription factor. *Genes & development* **12**, 2488-2498 (1998).

10. Seydoux G, Dunn MA. Transcriptionally repressed germ cells lack a subpopulation of phosphorylated RNA polymerase II in early embryos of Caenorhabditis elegans and Drosophila melanogaster. *Development* **124**, 2191-2201 (1997).

11. Powell-Coffman JA, Knight J, Wood WB. Onset of C. elegans gastrulation is blocked by inhibition of embryonic transcription with an RNA polymerase antisense RNA. *Developmental biology* **178**, 472-483 (1996).

12. Derry WB, Putzke AP, Rothman JH. Caenorhabditis elegans p53: role in apoptosis, meiosis, and stress resistance. *Science* **294**, 591-595 (2001).

13. Yasuda M, D'Sa-Eipper C, Gong XL, Chinnadurai G. Regulation of apoptosis by a Caenorhabditis elegans BNIP3 homolog. *Oncogene* **17**, 2525-2530 (1998).

14. Cizeau J, Ray R, Chen G, Gietz RD, Greenberg AH. The C. elegans orthologue ceBNIP3 interacts with CED-9 and CED-3 but kills through a BH3- and caspase-independent mechanism. *Oncogene* **19**, 5453-5463 (2000).

15. Ceol CJ, Horvitz HR. dpl-1 DP and efl-1 E2F act with lin-35 Rb to antagonize Ras signaling in C. elegans vulval development. *Molecular cell* **7**, 461-473 (2001).

16. Chi W, Reinke V. DPL-1 (DP) acts in the germ line to coordinate ovulation and fertilization in C. elegans. *Mechanisms of development* **126**, 406-416 (2009).

17. Schumacher B*, et al.* C-elegans ced-13 can promote apoptosis and is induced in response to DNA damage. *Cell Death Differ* **12**, 153-161 (2005).

18. Lee RY, Lobel L, Hengartner M, Horvitz HR, Avery L. Mutations in the alpha1 subunit of an L-type voltage-activated Ca2+ channel cause myotonia in Caenorhabditis elegans. *Embo J* **16**, 6066-6076 (1997).

19. An JH, Blackwell TK. SKN-1 links C. elegans mesendodermal specification to a conserved oxidative stress response. *Genes & development* **17**, 1882-1893 (2003).

20. Towers PR, Edwards B, Richmond JE, Sattelle DB. The Caenorhabditis elegans lev-8 gene encodes a novel type of nicotinic acetylcholine receptor alpha subunit. *J Neurochem* **93**, 1-9 (2005).

21. Wang BB, Muller-Immergluck MM, Austin J, Robinson NT, Chisholm A, Kenyon C. A homeotic gene cluster patterns the anteroposterior body axis of C. elegans. *Cell* **74**, 29-42 (1993).

22. Murakami S, Johnson TE. Life extension and stress resistance in Caenorhabditis elegans modulated by the tkr-1 gene. *Current biology : CB* **8**, 1091-1094 (1998).

23. Murphy CT*, et al.* Genes that act downstream of DAF-16 to influence the lifespan of Caenorhabditis elegans. *Nature* **424**, 277-283 (2003).

24. Hoogewijs D, Houthoofd K, Matthijssens F, Vandesompele J, Vanfleteren JR. Selection and validation of a set of reliable reference genes for quantitative sod gene expression analysis in C. elegans. *BMC molecular biology* **9**, 9 (2008).

25. Saijou E, Fujiwara T, Suzaki T, Inoue K, Sakamoto H. RBD-1, a nucleolar RNA-binding protein, is essential for Caenorhabditis elegans early development through 18S ribosomal RNA processing. *Nucleic acids research* **32**, 1028-1036 (2004).

26. Ichimiya H, Huet RG, Hartman P, Amino H, Kita K, Ishii N. Complex II inactivation is lethal in the nematode Caenorhabditis elegans. *Mitochondrion* **2**, 191-198 (2002).

27. Bowerman B, Eaton BA, Priess JR. skn-1, a maternally expressed gene required to specify the fate of ventral blastomeres in the early C. elegans embryo. *Cell* **68**, 1061-1075 (1992).

28. Kenyon CJ. The genetics of ageing. *Nature* **464**, 504-512 (2010).

29. Ludewig AH*, et al.* Pheromone sensing regulates Caenorhabditis elegans lifespan and stress resistance via the deacetylase SIR-2.1. *Proc Natl Acad Sci U S A* **110**, 5522-5527 (2013).

30. Larsen PL. Aging and resistance to oxidative damage in Caenorhabditis elegans. *Proc Natl Acad Sci U S A* **90**, 8905-8909 (1993).

31. Hunter T, Bannister WH, Hunter GJ. Cloning, expression, and characterization of two manganese superoxide dismutases from Caenorhabditis elegans. *J Biol Chem* **272**, 28652-28659 (1997).
